# Profiles of Burnout and Response to the COVID-19 Pandemic Among General Surgery Residents at a Large Academic Training Program

**DOI:** 10.1177/15533506221120145

**Published:** 2022-08-16

**Authors:** May-Anh Nguyen, Matthew Castelo, Brittany Greene, Justin Lu, Savtaj Brar, Emma Reel, Tulin D. Cil

**Affiliations:** 1 Division of General Surgery, Department of Surgery, 7938 University of Toronto, Toronto, Ontario, Canada; 2 Institute of Health Policy, Management and Evaluation, Dalla Lana School of Public Health, 7938 University of Toronto, Toronto, Ontario, Canada; 3 Temerty Faculty of Medicine, 7938 University of Toronto, Toronto, Ontario, Canada

**Keywords:** burnout, COVID-19, general surgery, internship and residency

## Abstract

**Background:**

COVID-19 has placed demands on General Surgery residents, who are already at high risk of burnout. This study examined the pandemic’s impact on burnout and wellness among General Surgery residents at a large training program.

**Methods:**

General Surgery residents at our institution completed a survey focused on self-reported burnout, mental health, perceptions of wellness resources, and changes in activities during the pandemic. Burnout was measured using the Maslach Burnout Inventory (MBI). Unsupervised machine learning (*k-means* clustering) was used to identify profiles of burnout and comparisons between profiles were made.

**Results:**

Of 82 eligible residents, 51 completed the survey (62% response rate). During COVID-19, 63% of residents had self-described burnout, 43% had depression, 18% acknowledged binge drinking/drug use, and 8% had anxiety. There were no significant differences from pre-pandemic levels (*p* all >.05). Few residents perceived available wellness resources as effective (6%). Based on MBI scores, the clustering analysis identified three clusters, characterized as “overextended”, “engaged”, and “ineffective”. Engaged residents had the least concerning MBI scores and were significantly more likely to exercise, retain social contact during the pandemic, and had less self-reported anxiety or depression. Research residents were overrepresented in the ineffective cluster (46%), which had high rates of self-reported burnout (77%) and was characterized by the lowest personal accomplishment scores. Rates of self-reported burnout for overextended and engaged residents were 73% and 48%, respectively.

**Conclusion:**

Surgical residents have high rates of self-reported burnout and depression during the COVID-19 pandemic. Clusters of burnout may offer targets for individualized intervention.

## Introduction

Burnout is a phenomenon that affects medical professionals at higher rates than the general population.^1^ Burnout is characterised by depersonalization, emotional exhaustion, reduced personal accomplishment, and is associated with increased risk of depression and suicide, decreased work productivity, and worse patient outcomes.^2-4^ This has garnered attention in the healthcare sector as a public health concern.^4^ General Surgery residents appear to be at particular risk of burnout – this may be a by-product of prolonged duty hours, chronic sleep deprivation, and patient care demands.^5,6^

The COVID-19 pandemic has exacerbated these existing issues among surgical trainees.^7,8^ In many training programs, General Surgery residents were redeployed to assist with COVID-19 care in intensive care units and provide procedural support, resulting in lost operating room experience.^9-13^ In addition to specific clinical challenges, the pandemic has affected physician wellbeing more generally through restrictions on personal activities, and fear of occupational infection and subsequent illness.^14-20^ While there have been efforts to study the impact of the pandemic on General Surgery resident burnout and wellness,^7,8^ there has not been a focused assessment on changes in personal activities such as exercise or alcohol use or the perceived effectiveness of the training program response. Furthermore, studies have tended to focus on earlier experiences with the pandemic. Finally, there is a lack of data specifically on Canadian General Surgery trainees. The Canadian residency training system lacks strict duty hour restrictions and this makes it challenging to extrapolate results of burnout studies from other countries.

In this survey study, we sought to quantify the prevalence of burnout during the pandemic and its effect on wellness among General Surgery residents in our large university-based training program. We also aimed to identify novel sub-groups characterized by clustering the burnout scales using machine learning techniques. Our objective was to acquire a deeper understanding of burnout in this population thereby identifying targets for future interventions.

## Methods

### Study Design

This was a cross-sectional survey performed within the General Surgery Residency Training Program at the University of Toronto. The Research Ethics Board at the University of Toronto reviewed and approved this study (#40135). This manuscript was prepared in accordance with recent calls for standardization among survey studies in surgery.^21^

### Participants

The General Surgery Residency Program at the University of Toronto is the largest surgical training program in Canada, comprised of approximately 10-15 clinical residents per postgraduate year (PGY), in addition to research residents completing full-time graduate degrees through the Surgeon Scientist stream. The survey was offered to all registered General Surgery residents as of February 16, 2021 through a provided URL. Two email reminders were sent 2 weeks apart. Two residents who developed the survey were excluded (MAN & BG).

### Survey Development and Characteristics

The objectives of the survey were to assess (i) resident perception on available wellness resources and educational initiatives, (ii) self-reported depression, anxiety, burnout, and concerning behaviours, (iii) formal burnout indices using a validated scale, and (iv) identify patterns and predictors of burnout in exploratory analyses. Where applicable, changes in response to the COVID-19 pandemic were assessed. These objectives were identified by program leadership with input from resident members (MAN & BG). The survey questions were developed and reviewed for clarity and sensibility. The survey was transferred to an online format (www.surveymonkey.com) and pilot tested prior to administration.

The survey consisted of 21 questions reporting on demographic data, postgraduate year, perceived importance of resident wellness compared to other required aspects of the program curriculum, perceived efficacy of wellness initiatives in the program and wellness resources available, and presence of regret pursuing General Surgery residency. Additionally, participants were queried on perceived burnout, self-reported presence of mental health illness (ie depression, suicidality, post-traumatic stress disorder), usefulness of wellness resources provided by the program, and changes in day-to-day activities prior to, and during the COVID-19 pandemic (Supplementary Material 1).

### Maslach Burnout Inventory

Formal presence of burnout during the COVID-19 pandemic was assessed with the Maslach Burnout Inventory Human Services Survey for Medical Personnel, hereafter referred to as the MBI.^22^ The MBI is a validated psychometric tool created to study burnout in medical professionals and consists of 22 statements describing job-related feelings (Supplementary Material 2), which are organized into three subcategories: emotional exhaustion (EE), depersonalization (DP), and personal accomplishment (PA). Participants score how often they experience various statements on a 7-point Likert scale ranging from 0 (never) to 6 (every day). Higher scores for emotional exhaustion and depersonalization, along with lower scores for personal accomplishment indicate higher degrees of burnout.

### Statistical Analysis

Participant characteristics and responses were tabulated. Responses to survey questions were presented as bar charts, comparing responses before and during the pandemic. Changes in the frequency of activities before and during the pandemic were presented as diverging stacked bar charts. Changes in response to the pandemic were assessed with McNemar’s test for binary survey responses, and the Stuart-Maxwell test for survey questions with more than two possible responses.

Mean scores for each MBI subscale were calculated and presented using box plots. We were interested a priori in differences in subscale scores by gender and level of training. These comparisons were made using t-tests, and ANOVA, respectively. Level of training was collapsed into junior resident (PGY-1 & PGY-2), senior resident (PGY-3 – PGY-5), and research resident (full-time graduate training). Finally, correlation coefficients were calculated for each MBI subscale.

We undertook an exploratory analysis of MBI burnout scores using unsupervised machine learning. *K-means* clustering is a machine learning method that assigns unlabelled data into a user-provided number of clusters by iteratively moving the cluster center to minimize the total within cluster variance.^23^ In short, subjects are randomly assigned to one of the desired number of clusters and the initial mean of each cluster computed. The algorithm re-assigns subjects to whichever cluster center they are now closest to, and the mean re-computed. This process continues iteratively until the cluster means stabilize and subjects are optimally assigned. *K-means* clustering was performed using participants’ scaled MBI responses as features. Cluster size was chosen by visually inspecting a scree plot for the ‘elbow’ of the curve (Supplementary Material 3),^24^ and based on previous literature describing burnout profiles using the MBI.^25^ We also evaluated performance by reporting the ratio of the within sum of squares to the total sum of squares. Participants were assigned to one of three clusters using the algorithm, and visualized on a biplot using principal component analysis (Supplementary Material 4). The three clusters of participants were examined and general trends described. Differences in survey responses and participant characteristics were assessed using t-tests for continuous variables and Fisher’s exact tests for categorical variables. When appropriate, categories were collapsed to reduce small cells. Given the exploratory nature of this analysis, adjustments for multiple comparisons were not made.^26^

All analyses were performed using R version 4.1.0 (R Foundation for Statistical Computing, Vienna, Austria). Missing data were managed with pairwise deletion. All statistical tests were two-sided, and a *P*-value ≤.05 was considered statistically significant.

## Results

### Participant Demographics

The survey was sent to 82 eligible General Surgery residents at the University of Toronto, and 51 completed the survey (62% response rate). Demographics are presented in Table 1. Most respondents were senior residents (21/51; 41%), followed by junior residents (18/51; 35%), and research residents (11/51; 22%). A majority of respondents identified as male (32/51; 63%).

**Table 1.** Participant Demographics.

Characteristic	Respondents (n = 51)
Year of training, no. (%)	
PGY1	6 (11.8)
PGY2	12 (23.5)
PGY3	9 (17.6)
PGY4	5 (9.8)
PGY5	7 (13.7)
Research	11 (21.6)
Prefer not to answer	1 (2.0)
Level of training, no. (%)	
Junior resident	18 (35.3)
Senior resident	21 (41.2)
Research	11 (21.6)
Prefer not to answer	1 (2.0)
Self-identified gender, no. (%)	
Male	32 (62.7)
Female	19 (37.3)

### Self-Reported Burnout and Mental Health

**Figure 1.**
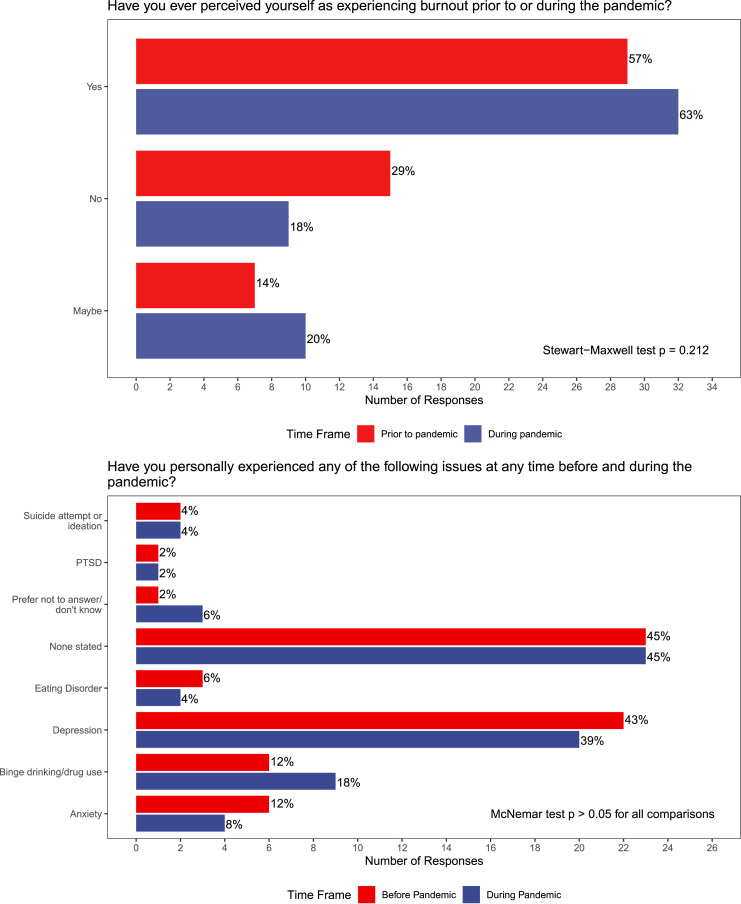
Self-identified rates of burnout prior and mental health concerns prior to and during the pandemic.

Prior to the pandemic, 57% of residents perceived themselves as having experienced burnout, compared to 63% during the pandemic (*P* = .212; Figure 1). Self-identified mental health concerns are also presented in Figure 1; 43% of residents described themselves as being depressed during the pandemic, 18% acknowledged binge drinking or drug use, and 8% had anxiety. Concerningly, 4% of residents reported suicide attempt or ideation during the pandemic. There were no significant changes from self-reported mental health concerns prior to the pandemic (McNemar test P > .05 for all comparisons).

### Resident Perceptions of Wellness and Educational Initiatives

**Figure 2.**
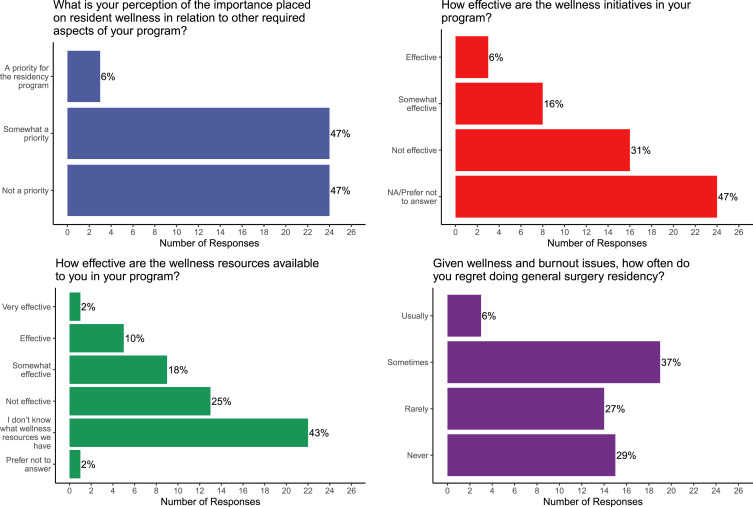
Perceptions of wellness resources, initiatives, and regret among General Surgery residents.

Overall, residents had poor perceptions of wellness initiatives in the program (Figure 2); 47% of surveyed residents felt wellness was not a priority and only 6% felt wellness initiatives were effective. A substantial proportion (43%) of residents did not know what wellness resources were available to them. Given wellness and burnout issues, 37% of respondents “sometimes” regretted choosing General Surgery, and 6% “usually” regretted their choice.

**Figure 3.**
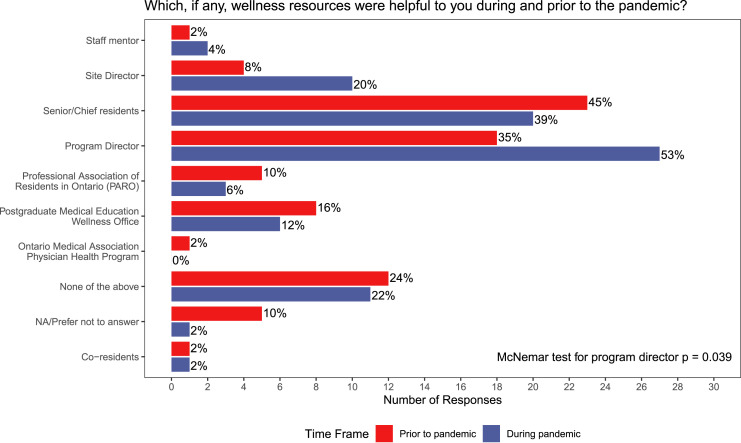
Helpful wellness resources prior to and during the pandemic. The only significant change in response to the pandemic was for the program director.

Resources identified as being the most helpful for wellness included the Program Director (35% prior to the pandemic vs 53% during; *P* = .039, Figure 3), and Senior/Chief residents (45% prior to the pandemic vs 39% during, *P* = .606, Figure 3). Fewer residents identified University-wide resources or professional organizations as being helpful during the pandemic, such as the Ontario resident union (6%), the Postgraduate Medical Education Wellness Office (12%), or the Ontario Medical Association Physician Health Program (0%).

### Change in Activities

**Figure 4.**
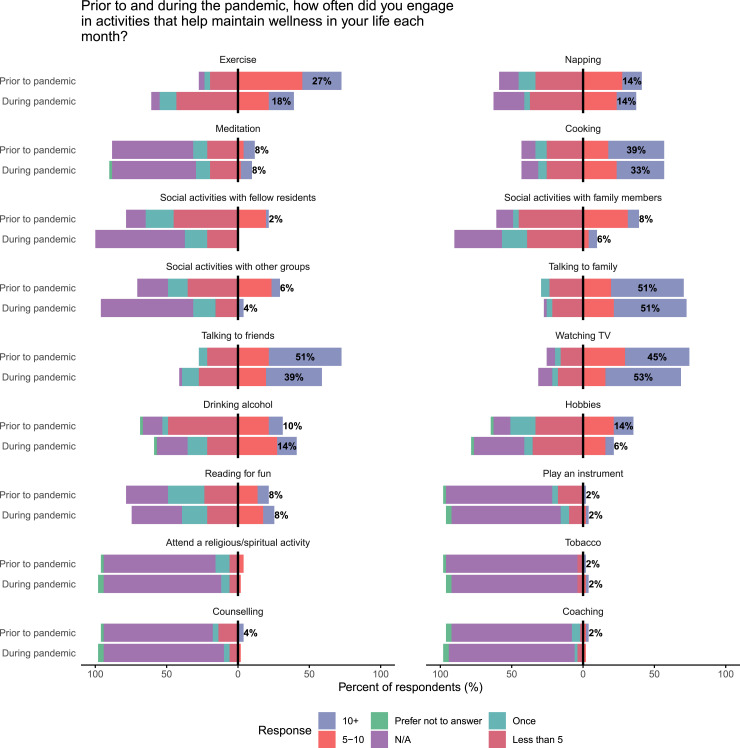
Changes in monthly activity frequency in response to the COVID-19 pandemic.

Residents were surveyed about changes in activities in response to the pandemic (Figure 4). There were nonsignificant trends towards lower social activities with fellow residents, family members, and other groups during the pandemic; 27% of residents exercised 10 times or greater per month prior to the pandemic, compared to 18% during the pandemic. During the pandemic, 14% of residents reported drinking alcohol 10+ times per month compared to 10% prior to the pandemic (P > .05). Overall there was very low engagement with religious/spiritual activities, counselling, or coaching, and low tobacco use (Figure 4).

### Maslach Burnout Inventory

**Figure 5.**
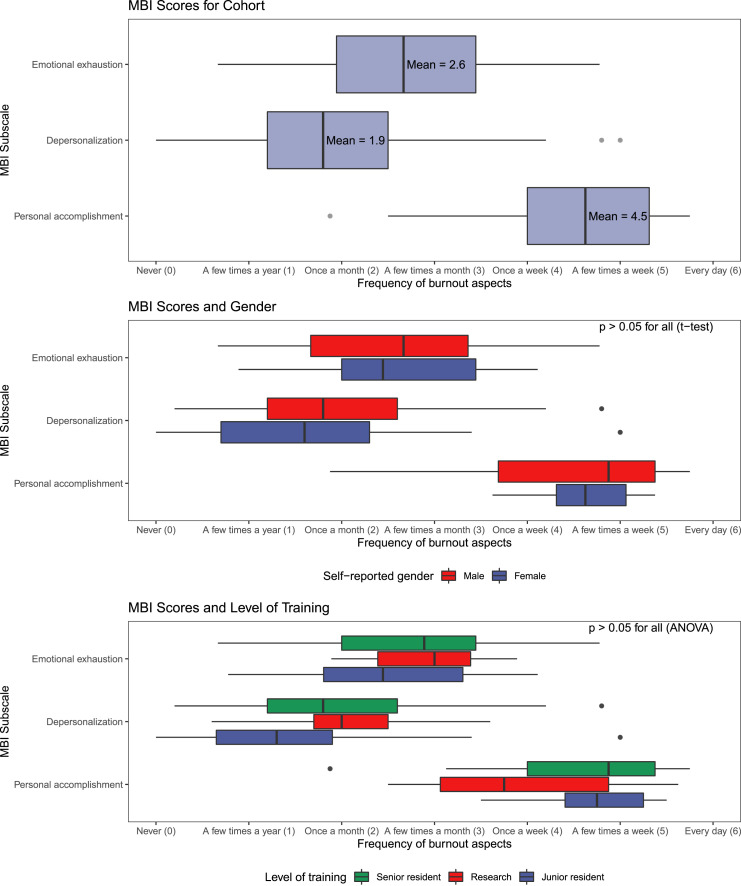
Maslach Burnout Inventory (MBI) subscale scores for the entire cohort of General Surgery residents, and stratified by gender and level of training.

Summary statistics for the three MBI subscales, stratified by gender and level of training are presented in Figure 5. Mean depersonalization (DP) occurred between a few times a year and once a month (mean score = 1.9/6). Mean emotional exhaustion (EE) occurred between once a month and a few times a month (mean score = 2.6/6). Residents scored feelings of personal accomplishment (PA) at a mean between once a week and a few times a week (mean score = 4.5/6). There were no significant differences in any subscale by gender or level of training (Figure 5). More detailed distributions are presented in Supplementary Material 5

### Clustering Analysis and Burnout Profiles

The *k-means* algorithm assigned participants into one of three clusters based on their MBI responses. The ratio of the within cluster sum of squares to the total sum of squares was .67, indicating 67% of the total variance was explained by the clustering. There were highly significant differences between the three clusters with regards to the mean EE, DP, and PA subscale scores (Table 2). Participants in the first cluster (n = 15) had the highest mean EE and DP scores, with high mean PA scores. We called this cluster “overextended”, in line with previous MBI research on burnout profiles.^25^ Twenty-three participants were assigned to cluster 2, which was characterized by the lowest mean EE/DP scores and the highest mean PA scores. This cluster was termed “engaged”. Finally, cluster three contained 13 participants, and had low mean EE/DP scores and low mean PA scores. This was consistent with an “ineffective” burnout profile.^25^

**Table 2.** Characteristics of Residents Assigned to Three Clusters by a *k-means* Algorithm. The Clusters Were Named post-hoc by Their Similarly to Previously Described Burnout Profiles from the Maslach Burnout Inventory.^25^

Characteristic or Survey Question	Overextended (n = 15)	Engaged (n = 23)	Ineffective (n = 13)	*P*-value
Emotional exhaustion, mean (SD)	3.69 (.53)	1.94 (.74)	2.71 (.81)	<.001
Depersonalization, mean (SD)	3.24 (.94)	1.28 (.63)	1.37 (.70)	<.001
Personal accomplishment, mean (SD)	4.55 (.71)	5.12 (.39)	3.37 (.70)	<.001
Level of training, no. (%)				
Clinical resident	12 (80.0)	20 (87.0)	7 (53.8)	.093
Research resident	3 (20.0)	3 (13.0)	6 (46.2)	
Gender, no. (%)				
Female	6 (40.0)	9 (39.1)	4 (30.8)	.870
Male	9 (60.0)	14 (60.9)	9 (69.2)	
Perception of wellness as a priority in the program, no. (%)				
A priority or somewhat a priority	3 (20.0)	15 (65.2)	9 (69.2)	.01
Not a priority	12 (80.0)	8 (34.8)	4 (30.8)	
Exercise frequency, no. (%)				
5+ per month	6 (40.0)	13 (56.5)	1 (7.7)	.018
Less than 5 times per month	9 (60.0)	9 (39.1)	10 (76.9)	
NA/Prefer not to answer	0 (.0)	1 (4.3)	2 (15.4)	
Talking to family, no. (%)				
5+ per month	8 (53.3)	22 (95.7)	7 (53.8)	.002
Less than 5 times per month	6 (40.0)	1 (4.3)	6 (46.2)	
NA/Prefer not to answer	1 (6.7)	0 (.0)	0 (.0)	
Talking to friends, no. (%)				
5+ per month	10 (66.7)	16 (69.6)	4 (30.8)	.044
Less than 5 times per month	4 (26.7)	7 (30.4)	9 (69.2)	
NA/Prefer not to answer	1 (6.7)	0 (.0)	0 (.0)	
Alcohol use, no. (%)				
5+ per month	9 (60.0)	9 (39.1)	3 (23.1)	.242
Less than 5 times per month	5 (33.3)	8 (34.8)	5 (38.5)	
NA/Prefer not to answer	1 (6.7)	6 (26.1)	5 (38.5)	
Self-reported burnout, no. (%)				
Yes	11 (73.3)	11 (47.8)	10 (76.9)	.166
Maybe	2 (13.3)	5 (21.7)	3 (23.1)	
No	2 (13.3)	7 (30.4)	0 (.0)	
Regret for general surgery, no. (%)				
Never or rarely	5 (33.3)	17 (73.9)	7 (53.8)	.076
Sometimes	8 (53.3)	6 (26.1)	5 (38.5)	
Usually	2 (13.3)	0 (.0)	1 (7.7)	
Self-reported depression or anxiety, no. (%)				
Yes	10 (66.7)	4 (17.4)	8 (61.5)	.004
No	5 (33.3)	19 (82.6)	5 (38.5)	
Binge drinking or drug use during pandemic, no. (%)				
Yes	3 (20.0)	4 (17.4)	0 (.0)	.286
No	12 (80.0)	19 (82.6)	13 (100.0)	

We examined demographic and survey response differences between the three clusters (Table 2). There were no significant differences by gender or level of training. The ineffective cluster contained the most research residents (46.2%) and the engaged cluster had the fewest, although this difference was not significant (13.0%; *P* = .093). Overextended residents were significantly more likely to perceive wellness as not a priority in the program (80.0% overextended vs 34.8% engaged vs 30.8% ineffective; *P* = .01). Engaged residents were significantly more likely to exercise (*P* = .018), talk to family (*P* = .002), or talk to friends (*P* = .044) 5 or more times per month compared to the other 2 clusters. Engaged residents were also significantly less likely to self-report depression or anxiety (66.7% overextended vs 17.4% engaged vs 61.5% ineffective; *P* = .004). Although there were trends towards overextended residents being more likely to use alcohol 5 or more times per month, “usually” regretting General Surgery, and binge drinking or using drugs, there were no significant differences between the three clusters with respect to these comparisons.

While the ineffective burnout profile is characterized by low scores across all three MBI subscales (including emotional exhaustion and depersonalization), we also found concerning characteristics in this group. Residents in the ineffective group were likely to report low exercise frequency (76.9%) and low frequency of talking to family (46.2%) or friends (69.2%). They also had the high rates of self-reported burnout (76.9%).

## Discussion

This cross-sectional survey study of 51 General Surgery residents at a large University-based program in Canada found substantial rates of self-described burnout and mental health concerns, poor perceptions of available wellness initiatives and resources, and a decrease in protective activities during the COVID-19 pandemic. Unsupervised machine learning identified three novel clusters of burnout based on the Maslach Burnout Inventory that may inform more individualized wellness interventions.

The physical and mental well-being of surgeons-in-training is integral for the effectiveness, longevity, and safety of any health care system. Given the hindrance of physician and trainee burnout in delivering quality patient care,^2-4^ various institutions around the world have sought to mitigate the incidence.^27,28^ Our results are largely consistent with COVID-19 experiences from other research groups. Poelmann et al^7^ reported MBI scores among 317 Dutch surgical residents in January 2020 compared to April 2020. There were no significant differences comparing pre- and post-pandemic scores, and their cohort similarly had high personal accomplishment scores.^7^ However, they were able to demonstrate a greater prevalence of burnout symptoms among residents that were redeployed to COVID-19 wards compared to non-redeployed residents. We did not explore this comparison in our cohort, as the prolonged and dramatic effect of COVID-19 on the Greater Toronto Area healthcare system by March 2021 precluded such a control group. The American College of Surgeons (ACS) completed a survey study in July 2020 including 465 resident members.^8^ This group showed high rates of self-reported depression (31%) and anxiety (61%), and those with higher levels of depression were less likely to report their institution had wellness resources.^8^

Our results have expanded on these findings by assessing residents’ perceptions of wellness resources in addition to their availability. Overall, our cohort of General Surgery residents did not feel wellness was a priority in their program, and the available resources/initiatives were not effective. In particular, wellness resources offered through professional associations were utilized least by our residents. This is consistent with the recent ACS survey, in which only 13% of residents reported using wellness resources offered by professional societies.^8^ These results suggest resources offered by large societies have low uptake among surgical residents and may not be well-suited to providing support during the pandemic. Conversely, residents in our cohort clearly indicated the Program Director and Senior/Chief residents were the most helpful resources available to them. There are several potential explanations why residents may prefer these resources, including ease of access, context familiarity, and perceived effectiveness. More work is needed to fully understand the factors influencing where surgical residents seek support so that resources may be effectively targeted and deployed. Although the quality of evidence is low,^29^ resident-led and program-led wellness initiatives have been shown to be effective in the literature.^30-32^ Training programs may have greater success in addressing burnout by developing tailored resources rather than relying on more generic professional societal initiatives.

Our study is one of very few in Canada to assess the prevalence of perceived and measured burnout in General Surgery residents before and during the COVID-19 pandemic, although our study does not contain data collected prior to the pandemic and is subject to recall bias. Self-reported burnout was high, with 63% of residents indicating they experienced burnout during the pandemic. A large survey study of 665 General Surgery residents in 2014 reported rates of burnout using the MBI of 69%.^33^ However, this sensitive definition of burnout was met if any MBI subscale score fell in the most concerning tertile. We did not a priori define burnout in this fashion, although the distribution of MBI scores were similar in our study (Supplementary Material 5).^33^ Indeed, the definition of burnout as a dichotomous outcome using the MBI is variable in the literature, and can result in widely different estimates. Hewitt et al^34^ applied a variety of accepted MBI burnout definitions using national survey data from General Surgery residents, and found proportions of burnout ranging from 3.2% to 91.4% in the same dataset. Given the difficulty in establishing a single cutoff for burnout, we undertook an unsupervised machine learning approach, considering the three MBI subscales in combination as unique “profiles” of burnout. The *k-means* clustering algorithm assigned participants into three clusters, which we characterized according to previously described MBI burnout profiles.^25^

Our analysis categorized 29% of respondents as overextended – residents with some signs of depersonalization or emotional exhaustion but retained high levels of personal accomplishment. In more traditional analyses, these residents may have been classified as having burnout.^33^ Reassuringly, 45% of residents were considered engaged. We hypothesize these profiles may be helpful in targeting wellness resources. Overextended residents in our cohort maintained some protective activities, such as exercise and social contact. These residents may respond to traditional wellness initiatives such as duty hour restriction enforcement, protected academic/personal time, and improvements in the clinical working environment (workspaces, reductions in ward demands, etc).^30^ The ineffective profile was characterized by low scores across all three subscales and represented the remaining 26% of residents. Rather than a burnout profile consisting of excessive demands (overextended), these residents were characterized by low levels of personal accomplishment/job satisfaction, and low rates of exercise and social contact. Although not statistically significant, there was a trend towards more research residents being classified as ineffective (46% vs 20% or less in the other groups, *P* = .093). Research residents take extended time away from clinical duties (2-4 years, typically) and complete advanced graduate degrees. It is possible they represent a distinct group within the resident cohort and may not respond to clinically focused wellness initiatives. Although the clinical demands of General Surgery residency are thought to be contributors to burnout symptoms, our results suggest research residents with no formal clinical duties are at particular risk. Indeed, while 51% of clinical residents were classified as engaged, only 25% of research residents were placed in this group. Further research is needed to explore these findings, which may be unique to a large academic training program – especially since these residents have been excluded from previous large survey studies.^33^

Clustering analysis has been used in some studies of burnout in medical professionals more broadly,^35,36^ and at least once among General Surgery residents. Kurbatov et al^37^ used *k-means* clustering on 53 General Surgery residents based on MBI scores and a number of other instruments. Similarly, three clusters were identified, which the authors corresponded to the underlying risk of burnout. Their analysis showed less clear separation when using principal component analysis compared to our results, which may be explained by the greater number of input features using by Kurbatov et al.^37^ This group also included scales measuring professional fulfillment, grit, occupational fatigue, and demographic factors in their clustering analysis.^37^ Taken together, these studies suggest that distinct profiles of burnout based on the MBI and similar scales are identifiable and interpretable. Given the previously mentioned challenges in setting cut-offs for dichotomizing burnout using the MBI,^34^ further work should continue to explore novel burnout profiles using clustering methods.

This study has several limitations. While our sample represents the largest General Surgery training program in Canada, these results may not be generalizable to residents in other countries, or to less academic training programs. In particular, 22% of our cohort was undertaking full-time graduate studies as research residents, which is relatively unique to the program.^38^ With 51 respondents, our study was limited in statistical power for key comparisons, including level of training, gender, and assigned burnout cluster. Survey studies are also limited by selection bias. There may be systematic differences between respondents and nonrespondents, and it is possible non-response is related to the outcome in question (burnout). Respondents may have different interpretations of survey questions, including terms such as “effective” and “usually”. Finally, our survey was cross-sectional and thus comparisons between pre-pandemic and during the pandemic were limited by recall bias.

Our study offers important insight into the burden of burnout and mental health concerns among General Surgery residents, a particularly vulnerable group of medical trainees. We have characterized changes in response to the COVID-19 pandemic and highlighted wellness resources that appear to resonate with trainees, and may deserve greater attention from program administrators. Finally, our clustering analysis identified distinct profiles of burnout among General Surgery residents that we hypothesize can be used to individualize interventions and may address some limitations in previous work utilizing the Maslach Burnout Inventory.

## Conclusions

In a single center cohort of General Surgery residents training at an academic program, this study reported high rates of self-reported burnout and depression that did not significantly worsen during the COVID-19 pandemic. Unique clusters of burnout were identified using machine learning methods that may offer targets for individualized wellness interventions.

## Supplemental Material

Supplemental Material - Profiles of Burnout and Response to the COVID-19 Pandemic Among General Surgery Residents at a Large Academic Training Program Click here for additional data file. Supplemental Material for Profiles of Burnout and Response to the COVID-19 Pandemic Among General Surgery Residents at a Large Academic Training Program by May-Anh Nguyen, Matthew Castelo, Brittany Greene, Justin Lu, Savtaj Brar, Emma Reel, and Tulin D. Cil in Surgical Innovation
