# Supplementary material for: Profiles of Burnout and Response to the COVID-19 Pandemic Among General Surgery Residents at a Large Academic Training Program
Source: Surg Innov. 2022 Aug 16;30(2):239–50. doi: 10.1177/15533506221120145 (PMC9382580; doi:10.1177/15533506221120145)

**Supplementary Material 1.** General Surgery Residency Wellness and Burnout Survey.

| Question Number | Question | Possible responses |
|---|---|---|
| 1 | Year of Study | a. PGY1<br>b. PGY2<br>c. PGY3<br>d. PGY4<br>e. PGY5<br>f. Research<br>g. Prefer not to answer |
| 2 | Gender | a. Female<br>b. Male<br>c. Other (free text response)<br>d. Prefer not to answer |
| 3 | What is your perception of the importance placed on resident wellness in relation to other required aspects of your program? | a. A priority for the residency program<br>b. Somewhat a priority<br>c. Not a priority<br>d. I don't know<br>e. Prefer not to answer |
| 4 | How effective are the wellness initiatives in your program? | a. Very effective<br>b. Effective<br>c. Somewhat effective<br>d. Not effective<br>e. I don't know what wellness initiatives we have<br>f. Other (free text response)<br>g. Prefer not to answer |
| 5 | How effective are the wellness resources available to you in your program? | a. Very effective<br>b. Effective<br>c. Somewhat effective<br>d. Not effective<br>e. I don't know what wellness resources we have<br>f. Other (free text response)<br>g. Prefer not to answer |
| 6 | Have you ever used the following wellness resources? (Tick all that apply) | a. Senior/Chief residents<br>b. Site Director<br>c. Program Director<br>d. General Surgery Wellness Lead<br>e. Postgraduate Medical Education Wellness Office<br>f. Professional Association of Residents in Ontario (PARO)<br>g. Ontario Medical Association Physician Health Program<br>h. Other (free text response)<br>i. Prefer not to answer |
| 7 | During the COVID-19 pandemic, which, if any, of the following wellness resources were helpful to you? Tick all that apply. | a. Senior/Chief residents<br>b. Site Director<br>c. Program Director<br>d. General Surgery Wellness Lead<br>e. Postgraduate Medical Education Wellness Office<br>f. Professional Association of Residents in Ontario (PARO)<br>g. Ontario Medical Association Physician Health Program<br>h. Other (free text response)<br>i. Prefer not to answer |
| 8 | During your residency training, have you personally experienced any of the following issues? (Select all that apply) | a. Depression<br>b. Suicide Attempt<br>c. Drug Use<br>d. Binge Drinking<br>e. Eating Disorder<br>f. I don't know<br>g. Other (free text response)<br>h. Prefer not to answer |
| 9 | During the COVID-19 pandemic, did you experience any of the following issues? | a. Depression<br>b. Suicide Attempt<br>c. Drug Use<br>d. Binge Drinking<br>e. Eating Disorder<br>f. I don't know<br>g. Other (free text response)<br>h. Prefer not to answer |
| 10 | The Professional Association of Residents of Ontario stipulates that every resident is entitled to 4 weeks of paid vacation each year in addition to 7 working days for educational purposes, a floating holiday, lieu day for working on statutory holidays, and 5 consecutive days during the winter holiday season. Check all that you have taken in the preceding academic year (July 1, 2019 – June 30, 2020) | a. 4 full weeks' vacation<br>b. 5 consecutive days of holiday vacation<br>c. Floating holiday<br>d. Lieu day<br>e. 7 full educational days<br>f. Other (free text response)<br>g. Prefer not to answer |
| 11 | If applicable, thinking back to earlier academic years, check all that you have taken. | a. 4 full weeks' vacation<br>b. 5 consecutive days of holiday vacation<br>c. Floating holiday<br>d. Lieu day<br>e. 7 full educational days<br>f. Other (free text response)<br>g. Prefer not to answer |
| 12 | During the COVID-19 pandemic, did you take your | a. Yes |

For Peer Review

| | | |
|---|---|---|
| | entitled leave? | b. No (free text response)<br>c. Prefer not to answer |
| 13 | During residency, have you ever taken sick days? | a. Yes<br>b. No, I have never required them<br>c. No, I was unable to<br>d. Other (free text response)<br>e. Prefer not to answer |
| 14 | During residency, have you ever taken lieu days? | a. Yes<br>b. No<br>c. Other (free text response)<br>d. Prefer not to answer |
| 15 | Prior to the Pandemic, how often did you engage in activities that help maintain wellness in your life each month? (10+, 5-10, less than 5, once, never) | a. Exercise<br>b. Napping<br>c. Meditation<br>d. Cooking<br>e. Social activities with fellow residents<br>f. Social activities with family members<br>g. Social activities with other groups<br>h. Talking to family<br>i. Talking to friends<br>j. Watching TV<br>k. Drinking alcohol<br>l. Hobbies<br>m. Reading for fun<br>n. Play an instrument<br>o. Attend a religious/spiritual activity<br>p. Tobacco<br>q. Counselling<br>r. Coaching<br>s. Other (free text response)<br>t. Prefer not to answer |
| 16 | Since the start of the Pandemic, how often do you engage in activities that help maintain wellness in your life each month? (10+, 5-10, less than 5, once, never) | a. Exercise<br>b. Napping<br>c. Meditation<br>d. Cooking<br>e. Social activities with fellow residents<br>f. Social activities with family members<br>g. Social activities with other groups<br>h. Talking to family<br>i. Talking to friends<br>j. Watching TV<br>k. Drinking alcohol<br>l. Hobbies<br>m. Reading for fun<br>n. Play an instrument<br>o. Attend a religious/spiritual activity<br>p. Tobacco<br>q. Counselling<br>r. Coaching<br>s. Other (free text response)<br>t. Prefer not to answer |
| 17 | Did you ever previously (prior to the Pandemic) perceive yourself as experiencing burnout? | a. Yes<br>b. Maybe<br>c. No |
| 18 | Do you currently (since the Pandemic) perceive yourself as experiencing burnout? | a. Yes<br>b. Maybe<br>c. No |
| 19 | How do you feel wellness education in residency training should be promoted? | a. Didactics<br>b. Off-site workshops<br>c. Online modules/videos<br>d. Podcasts<br>e. Program retreats<br>f. Team building exercises<br>g. Other (free text response)<br>h. Prefer not to answer |
| 20 | Given wellness and burnout issues, how often do you regret doing general surgery residency? | a. Never<br>b. Rarely<br>c. Sometimes<br>d. Often<br>e. Other (free text response)<br>f. Prefer not to answer |
| 21 | Do you have any further comments? | a. Yes (open free text)<br>b. No |

For Peer Review

**Supplementary Material 2.** Sample of Maslach Burnout Inventory Human Services Survey for Medical Personnel.

**MBI - Human Services Survey for Medical Personnel - MBI-HSS (MP)**:

I feel emotionally drained from my work.
I have accomplished many worthwhile things in this job.
I don't really care what happens to some patients.

Copyright ©1981, 2016 by Christina Maslach & Susan E. Jackson. All rights reserved in all media. Published by Mind Garden, Inc., www.mindgarden.com

For Peer Review

**Supplementary Material 3.** Scree plot to determine optimal cluster size during *k-means* clustering.

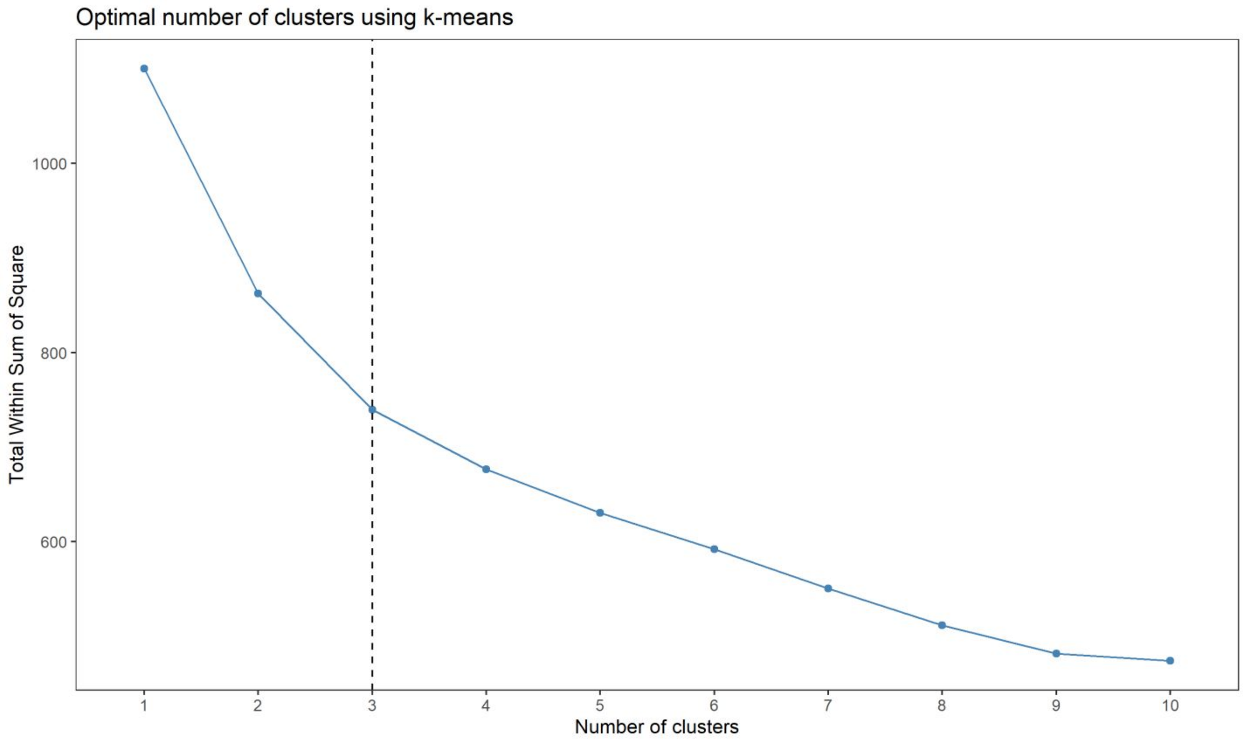


review

**Supplementary Material 4.** Biplot from *k-means* clustering with k = 3 based on the Maslach Burnout Inventory responses for 51 General Surgery residents. Respondents are presented in two dimensions using principal component analysis, with the first two principal components shown. Separation of the clusters is shown, with minimal overlap.

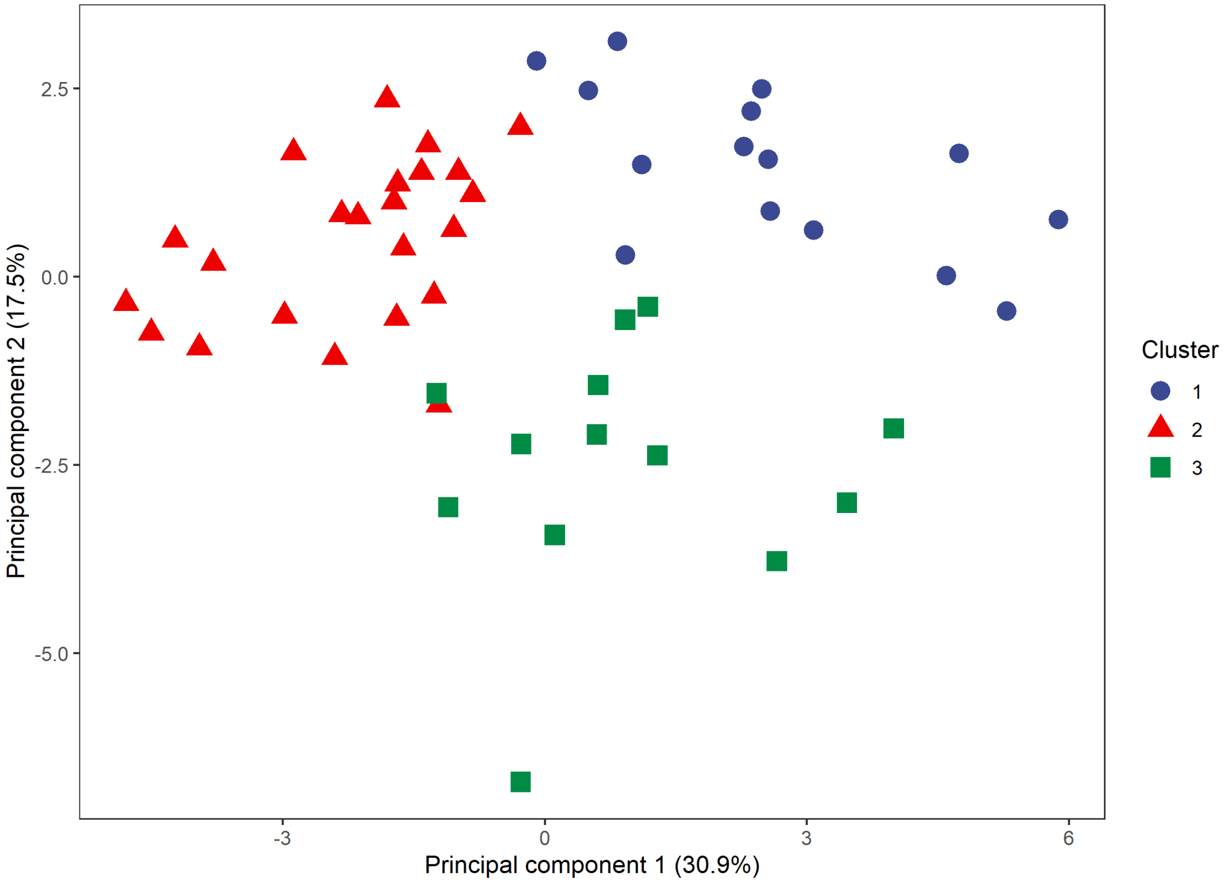

**Supplementary Material 5.** Histograms of MBI Subscales (total scores presented).

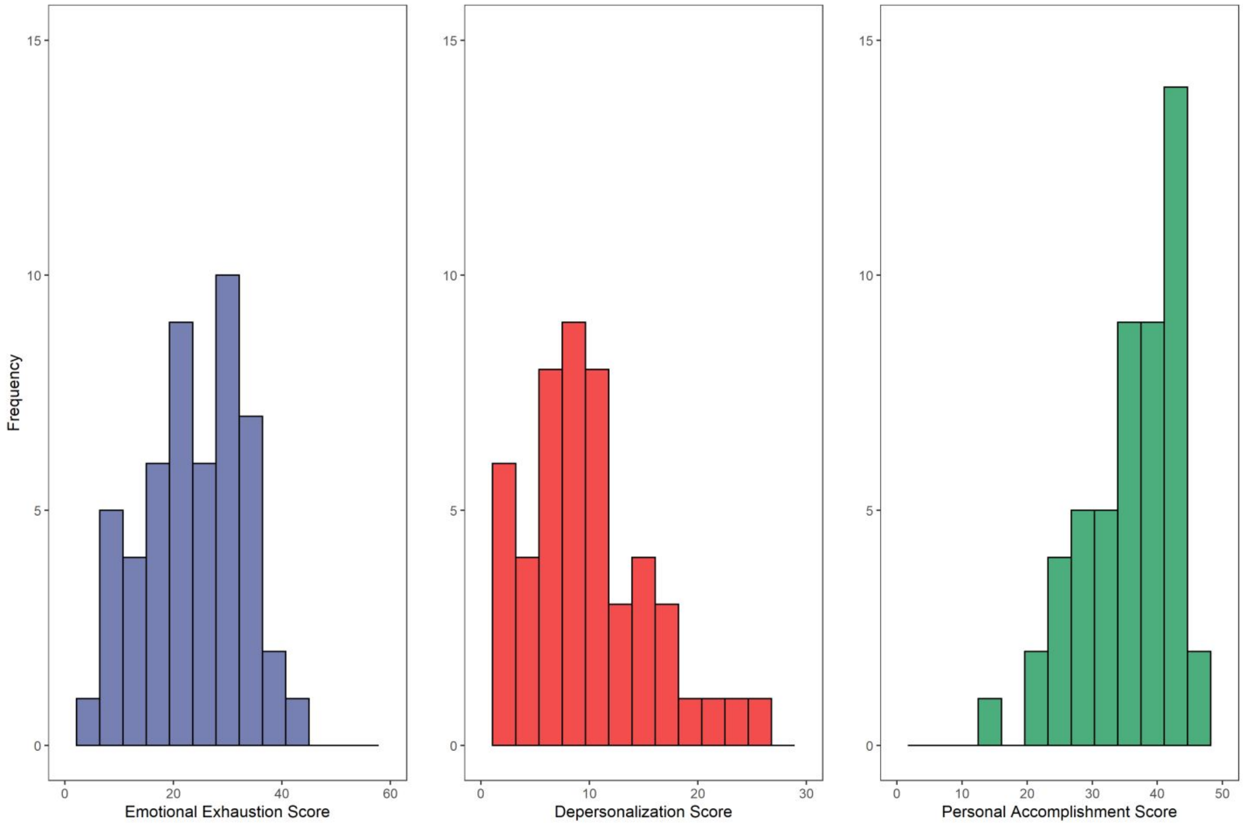


iew

**Supplementary Material 6.** Correlogram demonstrating correlation coefficients between the three subscales in the Maslach Burnout Inventory. Higher emotional exhaustion scores were positively correlated with higher depersonalization scores. Higher personal accomplishment scores were negatively correlated with the other two subscales.

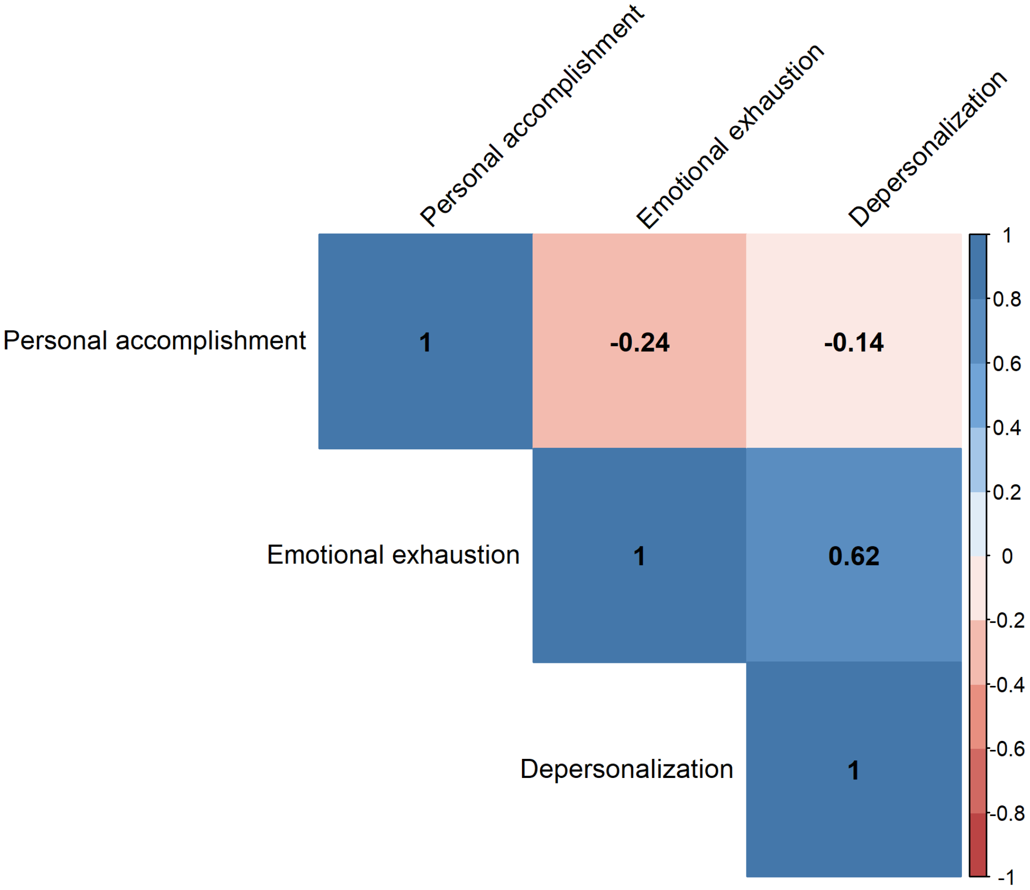

Supplement: Supplemental Material - Profiles of Burnout and Response to the COVID-19 Pandemic Among General Surgery Residents at a Large Academic Training Program [file sj-pdf-1-sri-10.1177_15533506221120145.pdf]
